# Which factors are related to Finnish home care workers’ job satisfaction, stress, psychological distress and perceived quality of care? - a mixed method study

**DOI:** 10.1186/s12913-020-05733-1

**Published:** 2020-09-23

**Authors:** Salla Ruotsalainen, Sami Jantunen, Timo Sinervo

**Affiliations:** 1 Finnish Institute for Health and Welfare, P.O. Box 30, 00271 Helsinki, Finland; 2 South-Eastern Finland University of Applied Sciences, P.O. Box 68, 50101 Mikkeli, Finland

**Keywords:** Home care, Self-organization, Job satisfaction, Quality of care, Mixed methods

## Abstract

**Background:**

The desire to increase the role of home care in Finland has created problems in home care work. Working conditions have deteriorated, the quality of care experienced is low, and staff members suffer from time pressure and stress, amongst other things. The aim of this article is to explore the challenges, stressors, teamwork and management factors that are associated with home care staff members’ well-being, job satisfaction and experienced care quality, and further, how staff members experience their work.

**Methods:**

A survey was sent to home care workers in two case organizations that participated in the study. In addition, semi-structured theme interviews with home care workers were conducted. The data from the survey was analysed using analysis of covariance, and interview data was analysed using the Grounded Theory-based method from Gioia et al.

**Results:**

Respondents of the survey and the interview participants were mainly female practical nurses. The results from the survey showed, for example, that time pressure was associated with higher stress and psychological distress, and interruptions were associated with lower job satisfaction and higher stress. In addition, variables related to teamwork, such as participative safety, were shown to explain the variation in quality of care. The analysis of the interview data further brought up dissatisfaction with management practices, which seems to have led to a decrease in job satisfaction. Exhaustion and strain were present among staff members, which originated from an insufficient number of carers.

**Conclusions:**

Current working conditions and work practices in Finnish home care are experienced stressful. The results from this study indicate that having more autonomy at work was associated with job satisfaction, according to both analyses. Team climate and idea implementation were related to quality of care. Therefore, increasing self-organizing team practices might be a possible development method for improving working conditions and staff members’ well-being. Implementing self-organizing team practices could possibly also attract employees to work in home care and prevent turnover.

## Background

In recent years, a service structure change has altered the care for older people in Finland. This structural change aimed to decrease the proportion of institutionalised care and significantly increase the proportion of home care provided for older people [1]. A similar trend in the care for older people has been evident elsewhere in Europe, since many countries have prioritised home care in their policies [2]. The objective of moving from institutional care towards more home-based care has been largely achieved in Finland, since institutional care beds (e.g. nursing homes and health centre inpatient wards) have been replaced with lighter solutions, like assisted living facilities and home care [3]. Furthermore, much emphasis has been placed on the development of home care through government key projects related to home care and informal care [4].

The desire to increase the role of home care has created new problems. The staffing of home care has not kept up with increases in home care clients [5]. Visits per day have increased throughout the 2000s, as has the complexity of home care clients’ problems. For instance, home care clients had more contacts with health care services compared to those older people living in assisted living facilities or other long-term care facilities [3]. Recent studies conducted among employees in the care for older people in Finland have indicated that working conditions in home care have deteriorated; home care workers experienced more time pressure and stress [6] compared to their counterparts in institutionalised care. In another Finnish study, the major problems in home care were time pressure, role conflict, working alone, interruptions, poor team climate and low organizational justice [7]. These earlier studies suggest that time pressure and other stressors, unjust management and problems in teamwork in particular might be the reasons for poorer well-being and dissatisfaction among Finnish home care workers. Interruptions at work and working alone have also been noted to burden the health care field [8] and home care [9] in other countries. Working alone was experienced stressful since work in home care is very independent and there might not be support available when needed [9].

Perhaps the most well-known theoretical model relating to the work environment and to employee well-being is the Job Strain Model, which is based on the idea that staff members with lower autonomy and higher demands at work have more health problems, or higher strain [10, 11]. Autonomy at work is also related to several positive outcomes, such as job satisfaction [12]. A second theoretical model is the Team Climate Model [13], which focuses on team characteristics. These characteristics are shown to be associated with team innovativeness [13] but also with several well-being issues such as burnout or emotional exhaustion [14, 15] and job satisfaction [16]. King et al. showed that a better climate for innovation in health care facilities reduced the negative effects of job demands [17]. The third model is the Organizational Justice Model. Different studies have shown that low organizational justice predicts low job satisfaction, higher stress, psychological stress, negative health effects [18], retirement intentions [19], sickness absence and several other negative outcomes, among others [15, 20, 21]. Furthermore, other studies conducted among care workers have shown that problems with teamwork and low supervisorial justice, for example, were associated with lower job satisfaction [12].

Not only well-being of employees but also the quality of care has decreased in Finnish services for older people, and especially in home care. In a Finnish study home care workers gave a lower grade for quality of care provided at their work unit than in other care facilities [22].

Unlike with employee well-being, there is far less evidence linking job characteristics, stressors, management or teamwork with quality of care. Previous studies conducted in the Finnish older people care context have used both objective (clinical quality outcomes, such as inadequate use of medication, pressure ulcers, use of restraints) and subjective outcomes (such as school grade for quality of care provided in the work unit) to measure care quality [22, 23]. Earlier studies have indicated that autonomy and job demands are strongly related to quality of care (drug use, pressure ulcers and restraint practices [23]) as well as quality of life [24], which is in line with Karasek model hypotheses. Results from the widely studied Magnet Hospital model showed that those hospitals that had adapted the Magnet principles and had a low organization, high decision-making authority in work units [25] as well as high autonomy [26] among nurses also rated the quality of care higher. Moreover, more supportive work environments were shown to have an impact on care quality [27, 28]. There is also evidence that team climate [29, 30] and organizational justice [15] are related to quality of care in primary care.

There is growing evidence that team autonomy or at least a high level of decision authority by the team is related to better outcomes of care on personnel (better job satisfaction, low stress) and on clients (better quality of care). The Magnet Hospital studies give support to this as well as the previously mentioned studies on team climate. The Dutch organization Buurtzorg, which provides home care services, has gained publicity with its promise of increased effectiveness and client and employee satisfaction [31], but these benefits have not largely been confirmed with scientific studies. In the Buurtzorg model of home care teams, there are no supervisors and teams can organize their work autonomously [31].

Initiating self-organizing teamwork practices has also shown some promising results elsewhere: A study by Maurits, et al. [32] showed that working in home care in a more self-organizing way was associated with higher job satisfaction. Moreover, in community mental health care, staff members were very satisfied with the increased independence and being able to influence their work more when self-organizing teamwork practices were initiated [33]. Increasing autonomy of teams has also shown to be associated with job satisfaction in other work sectors [34].

There is evidence that all the major models explaining staff members’ well-being have an effect on home care workers’ well-being as well as quality of care. However, there is not enough evidence of how these different models relate to each other, and which of these factors are most important.

### Context of the research

Home care in Finland refers to a combination of home help services and home nursing. Home help services include assistance with activities such as housing, personal care and other functions related to daily living specified in the Social Welfare Act [35]. Home nursing comprises health and medical care that is provided according to a treatment and care plan, or on a temporary basis, either at the patient’s home or in another place of residence [36]. Even if home care also includes home help services, it has become more medical and care-focused during the past years [37] and therefore other needs of the clients, such as housekeeping, have been disregarded. In addition to home help and home nursing, there are support services such as meals on wheels and cleaning services, and these very often need to be paid for out-of-pocket. In this study, from now on, we will use the term home care, since the case organizations in which this study was conducted provide home care that includes both home help services and home nursing.

Home care personnel in Finland mainly comprise practical nurses who have a three-year vocational qualification in social and health care [38]. Their main role is to provide daily care, but especially in home care their role can be broader and they can deliver medication, for instance. The role of a practical nurse is described in more detail elsewhere [19]. By approximately 12%, registered nurses comprise a significantly lower proportion of home care staff [39].

Finland has publicly-funded health and social care services where only a small proportion is produced by private service providers, except in assisted living, where more than half are private service providers (for-profit and not-for-profit). Municipalities are in charge of organizing health and social care services and mainly produce the services. The size of the municipalities can vary from a small rural town of 1000 inhabitants to a metropolitan area with approximately 500,000. In home care, the proportion of privately-produced care that municipalities purchase is only 0.4% of all home care [40, 41].

## Methods

### Aim and research questions

This study is a part of a larger project coaching home care teams towards self-organizing team practices. The aim of this article is to explore challenges, stressors, teamwork and management factors associated with home care workers’ well-being, job satisfaction and experienced care quality. Due to the scarcity of previous studies exploring home care workers’ well-being, job satisfaction and care quality, we wanted to include several predictors from the previously tested theories that have been shown to have an association with the outcome variables included in the present study. Further, we aimed to identify possible development needs of the work practices. The research questions for the study are:

1. Which teamwork factors, stressors and management related factors are associated with Finnish home care workers’

- job satisfaction
- stress
- psychological distress
- self-rated quality of care?

2. How do Finnish home care workers perceive their work?

### Study design and sample

In this cross-sectional study we used a mixed methods approach. We chose this approach for complementary purposes, in other words the two methods complemented each other [42]. The data for both quantitative and qualitative analysis was gathered in 2018 before interventions aiming to increase self-organizing work practices in the case organizations took place. A job satisfaction survey and semi-structured interviews for home care personnel were the two data sources for the present study and they are described in more detail later in the article. The survey and interviews were used as a preliminary benchmarking of well-being and for mapping the development needs among home care workers.

#### Study setting

The case organizations for the study were from two different locations in Finland. One case organization is responsible for providing home care in a large city in Southern Finland. However, only four districts of the organization were involved in the development project, including 22 teams. Another case organization comprised three home care teams from a smaller town, including more rural parts. Both case organizations are publicly funded and produce health and social care services themselves. In total, the case organizations had seven different districts and a total of 25 home care teams that participated in the study.

#### Measures

All variables used in the study are presented in Table 1. Outcome variables were job satisfaction, stress, psychological distress and quality of care. Job satisfaction was measured with one question (‘In general, I am very satisfied with my job’) and respondents were asked to evaluate this on a five-point Likert scale (1 – ‘strongly agree’ to 5 – ‘strongly disagree’) [43]. Stress was measured with one question: Do you currently feel stressed? (Stress means a situation when a person feels tense, restless, nervous or anxious, or is unable to sleep at night because his or her mind is troubled all the time) [44]. This was rated on a 5-point scale from ‘not at all’ to ‘very much’. To measure staff members’ psychological distress, a short version of the General Health Questionnaire (GHQ) was used [45]. Four items were selected: Have you recently lost a lot of sleep due to some worry? Have you recently been feeling unhappy and depressed? Have you recently felt constantly under strain? and Have you recently felt that you could not overcome your difficulties? The Cronbach’s alpha value for the four items was .83. Lastly, quality of care (QoC) was initially a sum of eight variables with a Cronbach’s alpha value of .84. In order to improve the consistency of the scale, one variable was removed. After removal, the alpha value was .87. The respondents assessed their co-workers’ 1) professional competence, 2) way of treating clients, 3) friendliness, 4) ability to answer clients’ requests for assistance, 5) ability to consider clients’ self-determination, 6) knowledge of issues related to the client, and 7) ability to consider the client’s next of kin. Here the rating was on a five-point scale from excellent (1) to bad (5) [46].

**Table 1:** Outcome variables and predictors used in the study

	Question	Scale	Mean (SD)	Cronbach’s α	Reference
Outcome variables					
Job satisfaction	Generally speaking, I am very satisfied with my job	1 ‘fully disagree’ - 5 ‘fully agree’	3.69 (1.05)		[43]
Stress	Do you currently feel stressed? Stress means a situation when a person feels tense, restless, nervous, or anxious, or is unable to sleep at night because his or her mind is troubled all the time.	1 ‘not at all’ - 5 ‘very much’	2.70 (1.10)		[44]
Psychological distress	Have you recently lost a lot of sleep over worry?; Have you recently been feeling unhappy and depressed?; Have you recently felt constantly under strain?; Have you recently felt you couldn’t overcome your difficulties?	1 ‘not at all’ - 4 ‘much more than usually’	2.14 (.74)	.83	[45]
Quality of care	How would you assess your co-workers’ …				
	1) professional competence, 2) way of treating clients, 3) friendliness, 4) ability to answer clients’ requests for assistance, 5) ability to consider clients’ self-determination, 6) knowledge of issues related to the client and 7) ability to consider the client’s next of kin.	1 ‘bad’ - 5 ‘excellent’	3.99 (.49)	.87	[46]
Predictors					
**Work stressors**					
	How often, during the past 6 months, have you felt disturbed, worried or strained due to …				
Working alone	Lack of consultation possibilities and collegial help?	1 ‘never’ - 5 ‘very often’	3.02 (1.08)		[47]
Interruptions	Constant interruptions and that work cannot be performed uninterrupted?	1 ‘never’ - 5 ‘very often’	3.02 (1.14)		[47]
Time pressure	I do not have enough time for patients; I do not have enough time to perform work properly; I only have time for the necessary tasks	1 ‘never’ - 5 ‘very often’	3.56 (.95)	.89	[48]
**Organizational justice**					
Relational justice	My supervisor treats his/her employees with kindness and consideration; My supervisor treats his/her employees respectfully; My supervisor considers his/her employees’ needs and listens to them.	1 ‘fully disagree’ - 5 ‘fully agree’	4.19 (.92)	.93	[49]
**Job control**					
Skill discretion	My work requires that I learn new skills	1 ‘fully disagree’ - 5 ‘fully agree’	4.36 (.75)		[10, 11]
Autonomy	At my work, I can make a lot of independent decisions	1 ‘fully disagree’ - 5 ‘fully agree’	3.75 (.83)		[10, 11]
Social support	When needed, I receive support from my 1. co-workers, 2. supervisors.	1 ‘never’ - 5 ‘always’	3.81 (.83)	.63	[10, 11]
**Teamwork**					
Participative safety	We have a “we are in it together” attitude; We keep each other informed about work related issues; We feel understood and accepted by each other; There are real attempts to share information throughout the practice	1 ‘fully disagree’ - 5 ‘fully agree’	3.68 (.73)	.83	[50]
Shared goals	Are you in agreement with the objectives set at you unit?; To what extent do you think your team’s objectives are clearly understood by other members of the team?; To what extent do you think your team’s objectives can actually be achieved?; How worthwhile do you think these objectives are to your team?	1 ‘very little’ - 5 ‘very much’	3.71 (.57)	.77	[50]
Idea implementation	New initiatives and ideas are taken up and assessed; At our work community, we can independently decide upon the implementation of a new development idea; At my work community, new thoughts and ideas are taken into action efficiently; Initiatives and ideas often lead to new practices, services and products in our organization	1 ‘fully disagree’ - 5 ‘fully agree’	2.91 (.78)	.81	[50]
Support for innovation	Search for new ways of looking at problems; Time taken to develop ideas; Cooperation in developing and applying ideas	1 ‘fully disagree’ - 5 ‘fully agree’	3.13 (.72)	.75	[51]

The predictors used in the study were chosen based on the three most well-known theories related to employee well-being (the Job Strain model, Team Climate Inventory and the Procedural Justice model). Our aim was to explore the associations between the outcome variables and predictors related to these models by using all the predictors in the same model to detect the most significant predictors. The predictors were categorised under the theoretical models. The first category was organizational justice, including a predictor of relational justice [49], the second category was teamwork, which included participative safety, shared goals and idea implementation predictors from the Team Climate Inventory [50] and support for innovation from Innovativeness scale by de Jong and den Hartog [51]. The third category was based on the Demand and Control model [10, 11] including predictors of social support, skill discretion and autonomy at work, and three predictors that have been identified as stressors in home care and care work by earlier studies [7–9]: working alone, interruptions at work [47] and time pressure [48]. All measures used in the survey are free to use for research purposes and do not require any license payments.

#### Data collection

The contact details of the home care workers who worked in the participating districts and teams were provided for us. A postal survey was sent to all home care workers of the participating teams in the two case organizations (*N* = 179). The number of staff members per team varied from three to 16. Teams were voluntarily able to take part in the project and to take a job satisfaction survey. The survey was aimed at permanent staff or substitutes who had worked in their current team for more than 6 months. The staff members were able to answer the survey in Finnish or Swedish (due to bilingualism of one municipality), and the response period was between April and May 2018. Reminders prompting participants to respond to the survey were sent twice: the first 3 weeks after the first survey round, and the second 3 weeks after the second survey round. Supervisors were asked to remind staff members to answer the survey.

#### Statistical methods

The survey data was analysed using analysis of covariance. First we used univariate analysis to analyse each predictor separately with each outcome variable to detect significant associations. Then analysis of covariance was performed with those predictors that were significantly associated with the outcome variables. Predictors with low association with the outcome variable were removed from the models stepwise, by first removing the one with the lowest association, then the second lowest and so on, until only the significant ones were left in the model. In total, this was performed for four separate models where job satisfaction, stress, psychological distress (GHQ) and quality of care were the outcome variables. Age and sex were adjusted for in all the covariance models. Significant associations for both univariate and multivariate associations are shown in Table 2. Statistical analyses were performed with SPSS version 25.

**Table 2:** Results from the analysis of covariance showing both univariate and multivariate associations for each outcome variable

	Model 1		Model 2		Model 3		Model 4	
	Job satisfaction		Stress		Psychological distress		Quality of Care	
	Univariate (F, p)	Multivariate (F, p)	Univariate (F, p)	Multivariate (F, p)	Univariate (F, p)	Multivariate (F, p)	Univariate (F, p)	Multivariate (F, p)
Working alone	3.28,*	0.02, ns	3.41, *	0.11, ns	5.11, **	5.64, *	1.16, ns	…
Interruptions	6.66,**	15.01, **	6.76, **	7.10, **	6.12, **	3.66, ns	2.64, *	1.88, ns
Time pressure	3.29, **	2.16, ns	4.10, **	6.69, *	4.36, **	9.84, **	1.48, ns	…
Relational justice	3.13,**	2.97, ns	2.50, **	1.03, ns	3.63, **	5.29, *	1.38, ns	…
Skill discretion	0.33, ns	…	0.81, ns	…	0.25, ns	…	1.49, ns	…
Autonomy	2.58,*	10.01, **	1.46, ns	…	2.97, *	0.94, ns	1.31, ns	…
Participative safety	2.62,**	3.42, ns	1.30, ns	…	1.18, ns	…	5.22, **	21.35, **
Shared goals	2.75, **	1.71, ns	1.63, ns	…	0.73, ns	…	4.49, **	4.39, *
Social support	6.27, **	0.47, ns	3.70, **	0.20, ns	4.18, **	0.20, ns	5.83, **	0.34, ns
Support for innovation	3.38, **	0.08, ns	2.05, *	0.11, ns	2.38, **	1.15, ns	4.47, **	0.47, ns
Idea implementation	3.07, **	10.97, **	2.19, *	7.08, **	1.48, ns	…	6.25, **	16.72, **
		Adjusted R^2^ .34		Adjusted R^2^ .31		Adjusted R^2^ .29		Adjusted R^2^ .46

*p < 0.05

**p < 0.01

… Not tested in the final model due to non-significant univariate association

### Semi-structured theme interviews

Our material was gathered by conducting semi-structured theme interviews. A semi-structured interview has predetermined themes, and they can be modified or additional themes and questions can be included depending on what is appropriate for a particular interview [52]. The use of semi-structured theme interviews were considered appropriate for the purposes of this study, because semi-structured theme interviews have been found to be suitable in situations where a study focuses on the meaning of particular phenomena to the participants and where individual perceptions of processes within a social unit are studied [52].

The themes of the interviews selected for this particular study included: 1. Job satisfaction, 2. Leadership and management, 3. Client work and client satisfaction, 4. Teamwork and its functioning, and 5. Trust. The themes were chosen to gain as comprehensive understanding of the case organizations’ home care, and its functioning, as possible. Even though the themes were predetermined, it should be pointed out that these themes did not restrict the emergence of any other theme considered important by the interviewees. In other words, the structure of the interviews was not restricted to issues only emerging from the five themes. The interviewees were able to freely discuss any topic they found relevant in relation to their work.

We interviewed 15 employees in 2018. Interviews lasted from 45 to 60 min and they were tape-recorded with permission from the interviewees and transcribed verbatim by a professional service firm. Interviewees were practical nurses (*n* = 14) with one registered nurse. All the participants were female. The anonymity of the participants was secured so that no individual is identifiable from the analysis. The interviews were carried out in Finnish, which is the interviewees’ native language, and then translated into English when writing the research report.

#### Analysis of data

Since our focus was on exploring the perceptions of home care workers related to their work, we decided to use interpretive and inductive research methods. In this study, a Grounded Theory approach was chosen because [53] it has been argued to be especially suitable in research areas where there is not enough knowledge or to which there is a need for new points of view [54].

In this paper we have adopted a Grounded Theory-based method, introduced by Gioia et al. [55] for the identification of essential conceptual categories. This method, used particularly in organizational studies, comprises first and second order analyses. The first order analysis captures salient findings from the interviews, with an attempt to adhere faithfully to informant terms [55]. This process has similarities with the process of open coding, as described by Strauss and Corbin [53]. In this step, we captured all portions from the interview that seemed important to the interviewees, using words that the interviewees had originally used themselves. In the second order analysis the focus of analysis shifts to condensing the identified first order concepts into a more manageable number. This is first done by seeking similarities and differences among the many categories (similar to Strauss and Corbin’s [53] notion of axial coding) and then asking whether the emerging themes suggest concepts that might help us describe and explain the phenomena we are observing [55]. Once a workable set of themes and concepts is in hand (and the culmination of the theme and concept development process leads to what Glaser and Strauss [56] termed ‘theoretical saturation’), we distilled the emergent second order themes further into second order ‘aggregate dimensions’, as suggested by Gioia and his colleagues [55].

Even though our qualitative analysis has been inductive, allowing any kind of concept or aggregate dimension to emerge, we have made minor adjustments to the wording of some of the second order themes and aggregate dimensions at the end of analysis. The purpose of these adjustments has been to improve the opportunities to compare qualitative and quantitative research results. Although the aggregate dimensions that emerged in this analysis resulted in being notably similar with the outcome variables of the quantitative analysis, the second order concepts and aggregate dimensions have emerged without forcing the results beforehand with predefined codes. Our qualitative analysis has been inductive rather than deductive by nature.

## Results

### Survey

#### Sample characteristics

The total number of participants was 121 and the response rate was 67%. Thirteen people responded to the survey in Swedish and 108 in Finnish. The mean age of the participants was 40.6 years, ranging from 19 to 65 years. Only two of the participants were male. The participants were mainly practical nurses (*N* = 104, 86%), and registered nurses comprised 12% of the sample. Only one participant reported being in a managerial position. The educational background of the respondents was mainly (81%) vocational training (licensed practical nurse or similar), and 12% had a degree from college or a university of applied sciences.

#### Analyses of covariance

**Fig. 1:**
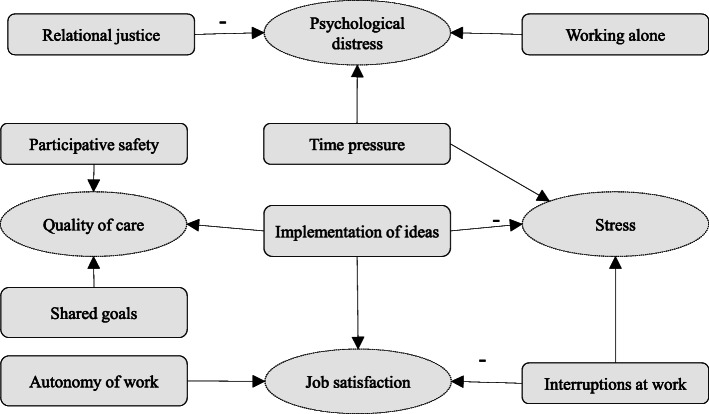
Associations from the covariance analyses models. Outcome variables are illustrated with a dashed line circle and predictors with squares.

The results from the univariate and covariance analyses are presented in Table 2 and Fig. 1. Analysis of covariance showed that interruptions, autonomy and idea implementation remained significant when all the variables were tested in the same model with job satisfaction (Table 2, model 1). Therefore, the predictors from the Demand-Control model were partly seen to explain job satisfaction as well as one predictor of the teamwork factors. With regard to stress, again the predictors from the Demand-Control model (interruptions at work and time pressure) and one teamwork factor (idea implementation) were associated with higher stress in multivariate analysis (model 2). The following predictors were associated with psychological distress: working alone and time pressure from the Demand-Control model and relational justice from the Organizational Justice model (model 3). Lastly, participative safety, shared goals and idea implementation of the Teamwork predictors were related to quality of care (model 4). Adjusted R squared values varied from .29 to .46 in the final models. These values could be considered to explain the variation in the outcome variables fairly well.

### Theme interviews

**Fig. 2:**
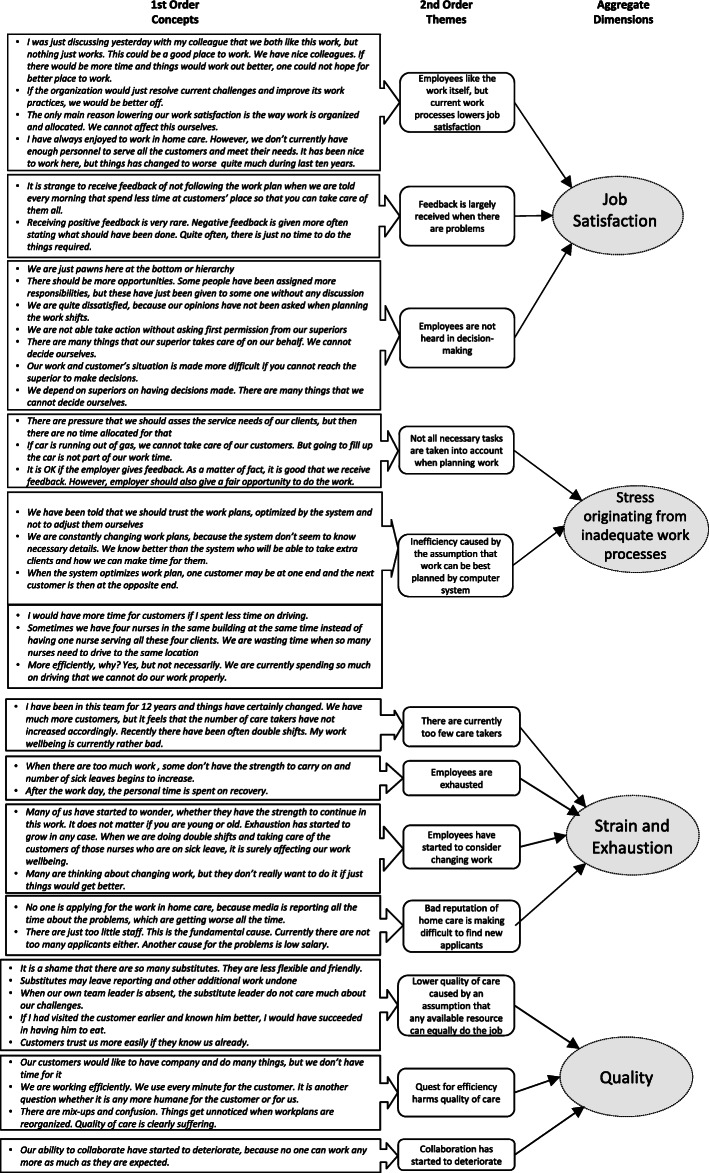
Coding process showing the formation of concepts, themes and aggregate dimensions.

As a result of following the Gioia methodology [55], we formed a graphical data structure showing how the analysis progressed from the data to the themes and aggregate dimensions (Fig. 2). This provides the reader with an opportunity to look at the grounded theory model and see how the essential concepts, themes and/or dimensions contained in the data structure were formed and how they are related to each other.

Our analysis revealed that although home care workers liked their work, current work processes and management styles seemed to lower their work satisfaction and increase stress. Home care workers felt that they were just pawns at the bottom of the hierarchy, being largely unable to influence the way work is planned. These feelings seem to have lowered home care worker’s job satisfaction. The staff members also felt that the way work was organized did not adequately help them to accomplish their objectives. As some examples, the desire to schedule work efficiently in a centralised manner seemed to have created problems on a practical level, largely because it is not possible for resource planning systems to know all the subtle details related to the work to be done. As a result, staff members felt that they were always in a hurry, spending too much time driving from one place to another and spending too little time with the clients.

The current situation in home care seems to make staff members strained and exhausted. This has led many of them to consider whether they had the strength to continue if their situation did not improve. Exhaustion seemed to have increased the number of periods of sick leave, which has increased the workload for those still at work. Furthermore, home care as a workplace has started to gain a bad reputation, which has made it more difficult to recruit staff.

Home care workers felt that there is just not enough time to conduct their work the way they wanted to. Another problem that appeared to bother both staff members and clients was that clients were rarely seen by the same carer. This would suggest that there is an unarticulated assumption believing that it is relatively irrelevant which carer is caring for the client. The conducted interviews suggest that such an assumption may be false. Several carers highlighted the benefits of knowing the clients deeply and the problems with using substitute carers. Some of the home care workers have also felt that, due to the stressful and exhausting work conditions, their ability to collaborate has started to deteriorate. These observations seem to have led to lower quality of care.

## Discussion

This study aimed to explore factors that are associated with Finnish home care workers’ well-being and perceived quality of care using three of the most well-known theoretical models related to employee well-being, teamwork and organizational justice. Furthermore, we wanted to understand how staff members perceive their work and other underlying factors that could disturb and stress the staff members and could be the potential causes of the stressors.

Previous studies have revealed problems among home care personnel with job characteristics [6, 7] and the results from our study support these findings. Our results highlight several factors that are shown to negatively influence home care workers’ well-being.

Both qualitative and quantitative analyses show similarities in terms of stress factors. The survey showed that time pressure was associated with higher psychological distress and stress, whereas the interviews revealed that there are currently too few home care workers, which has caused exhaustion. In an attempt to deal with this situation, the management teams of home care organizations seem to have often tried to optimise the use of existing staff members. Unfortunately, planned work processes have often turned out to be less than ideal on a practical level. The interviews revealed that problems with following processes and the inability to sufficiently influence the way work is conducted have increased home care workers’ stress. This is in line with the quantitative finding indicating an increase in stress when the implementation of own ideas is not supported. The results, which suggest that organizational justice and working alone are related to distress, are also well in line with earlier results [10, 20]. What is noteworthy is that autonomy of staff members, which is an important part of the demand-control model, was not related to psychological distress or experienced stress. This may be due to the characteristics of home care work, where work is highly independent. Autonomy at work was, however, related to job satisfaction.

Regarding job satisfaction, similarities with both qualitative and quantitative analysis were also found. Less autonomy at work and less idea implementation were shown to negatively influence job satisfaction, whereas the staff members stated in the interviews that they are not able to take action on their own and that there is a lack of opportunities to make decisions. The association between decision latitude and job satisfaction among nurses is widely known from the previous literature [57]. In the univariate analyses, relational justice was associated with job satisfaction but after adding other variables to the model, it lost its significance. In the qualitative analyses, management practices came up as home care workers stated being dissatisfied with their supervisors who did not listen to staff when making decisions. It is essential to enhance relational justice at workplaces, since it can be an important predictor of poorer self-rated health and sickness absence [18]. In particular, when aiming to increase self-organizing team practices, the role of management should be supportive rather than ‘micromanaging’. These findings could be connected to the implementation of ideas as well. In quantitative analyses, idea implementation was associated with job satisfaction, stress and quality of care. A similar conclusion seemed to arise from the interview analyses where employees’ experiences of not being heard in the decision-making process was linked to a decrease in job satisfaction. Moreover, one factor that might explain the dissatisfaction related to management in the present study could be that management practices varied across home care teams. Some supervisors were too controlling and the staff members were not able to take actions on their own, whereas other supervisors were absent and the staff members were not able to reach them. The findings suggest that job satisfaction could be increased by allowing home care workers to influence their job and increasing their ability to manage their working days. Autonomy of home care work is presumably one of the appealing characteristics of the job. A study conducted in Sweden also indicated that nurses working in community care appreciated the independence of their work and the ability to use their knowledge and skills [58]. However, due to current issues in Finnish home care, this appeal of autonomy at the employee level might no longer be true and further, problems with recruitment also arose from the findings of our study. Therefore, increasing the autonomy of home care teams could be one possible solution when aiming to attract more staff members to home care, prevent turnover and increase employee well-being.

Interruptions were also one of the stressors that was detected to be negatively associated with job satisfaction in the quantitative analyses. Interruptions can be a great source of frustration and increase the chance of errors [59]. Similar to the findings by Wilkes, et al. [60], our study identified an association between interruptions and stress. Interruptions have also been found to be a cause of other negative outcomes such as cognitive exhaustion [60] and a risk for patient safety [61]. Even though interruptions were strongly associated with stress and job satisfaction, the staff members did not highlight them significantly in the interviews. Still, their influence should not be neglected in home care work where work places and situations change frequently and care staff mainly work independently without other people to supervise or ‘double check’ their work.

Despite all the stressors arising from the results, there were some encouraging and positive aspects as well. The survey results showed that participative safety was associated with better quality of care and in the interviews the participants highlighted that they fully trusted their colleagues. This needs to be considered important, since trust can have a positive influence on employee well-being and job satisfaction [62]. Trust, however, was not covered in the survey and therefore we cannot draw very robust conclusions as to what extent it had an impact on the employee well-being or care quality. Moreover, home care workers stated in the interviews that they liked their work and based on their views the problems seemed to be more related to the processes and ways of organizing the work, rather than to the care work itself. Based on this finding it would be important to modify the work processes according to the suggestions from the staff members, for instance by allowing them to implement their ideas in improved practices. This could further increase their experience related to fair management, which could then lead to higher job satisfaction or less sickness absence [18].

The findings from the quantitative analyses regarding quality of care seemed to support the findings from the interviews to some extent; variables related to teamwork were important predictors in the univariate analyses and in the final model, participative safety, shared goals and idea implementation still remained significant. The relationship between the factors of teamwork and quality of care is an interesting finding, since there is very limited evidence of the relationship between team climate and quality of care [29, 30]. There is, however, evidence that team climate is associated with team effectiveness [63]. In the present study, deterioration in collaboration between home care workers seems to have led to a decrease in care quality. It is interesting that in our study, factors explaining the quality of care were all related to teamwork, whereas earlier findings from hospitals and institutional care of older people are more related to stress and autonomy of staff members [23]. However, in the Magnet hospital study, both flat organization and decision-making authority at the work unit level were also highlighted [25].

The results from our study could be interpreted in a way that using self-organizing teams as a development method could have potential in Finnish home care services. Staff members stated that they were better at planning their daily work schedules than the planning system they used at work. The interviews showed that the team members felt they had very little opportunity to make decisions, and they had to ask permission from supervisors, even for small things. By giving staff the opportunity to put their ideas into practice, there could be a potential effect on well-being and even better care quality. The importance of idea implementation could also indicate that staff, in general, want to be able to and are capable of participating in the development of their job. Based on the findings of our study, giving staff a chance to implement better practices at work could have several benefits in terms of their well-being. It is worth noting that home care workers themselves know their work best.

### Limitations

There were several limitations to this study. First of all, our sample size was very small. Furthermore, no other professional groups working in home care (such as physiotherapists or occupational therapists) were included in this study; therefore, this study provided only the views of practical nurses and registered nurses. Due to issues with data quantity, a mixed-methods approach was used in the study in order to gain richer data. Secondly, supervisors in the participating teams might have been aware of those who had not responded to the survey in the first round since they were in charge of delivering the surveys at the work units to those individuals who did not respond. This could have led to staff feeling compelled to respond to the survey, even if participation was on a voluntary basis. Thirdly, no causal relationship can be drawn from the results of the study, since a cross-sectional design was used. Therefore, no thorough conclusions can be made from the results since a cross-sectional study design can only provide a snapshot of a current situation and cannot take into account all factors that can influence the findings. Fourthly, the themes of the interviews could have been selected more according to the questions in the survey. For example, trust was not covered in the questionnaire even though it could have provided more robust conclusions related to home care staff members’ well-being or job satisfaction when combined with the qualitative analyses. However, the themes of the interviews were only loosely predetermined and similar topics arose from the analyses of the interview data as from results of the covariance analyses.

## Conclusions

Current working conditions and work practices in Finnish home care are considered stressful. For instance, staff members suffer from time pressure and interruptions. In addition, what transpired from the interviews was that high levels of absence of staff members led to strain and exhaustion among other staff members. In addition, the results showed that having more autonomy at work, i.e. being able to influence one’s job, was associated with job satisfaction. The survey results further showed that team climate and innovativeness of teams were related to quality of care. The results of this study could be interpreted to mean that implementing self-organizing team practices might be a possible development method to improve the situation of home care workers. If staff members could influence their work, this could have a positive impact on their well-being, through improved job satisfaction, for instance. On a broader scale, implementing self-organizing team practices could potentially increase the attractiveness of work in home care and prevent turnover of home care staff.

## Data Availability

Both quantitative and qualitative datasets used and/or analysed during the current study are available from the corresponding author on reasonable request. Note that the transcribed qualitative interview data is in Finnish.

## Abbreviations

GHQ: General health questionnaire
QoC: Quality of care
